# A Virulence Associated Siderophore Importer Reduces Antimicrobial Susceptibility of *Klebsiella pneumoniae*

**DOI:** 10.3389/fmicb.2021.607512

**Published:** 2021-01-28

**Authors:** Robeena Farzand, Kumar Rajakumar, Michael R. Barer, Primrose P. E. Freestone, Galina V. Mukamolova, Marco R. Oggioni, Helen M. O’Hare

**Affiliations:** ^1^Leicester Microbial Sciences and Infectious Diseases Network, Department of Respiratory Sciences, University of Leicester, Leicester, United Kingdom; ^2^Department of Microbiology, Kohat University of Science and Technology, Kohat, Pakistan; ^3^Department of Genetics and Genome Biology, University of Leicester, Leicester, United Kingdom; ^4^Department of Molecular and Cell Biology, University of Leicester, Leicester, United Kingdom

**Keywords:** *Klebsiella pneumoniae*, yersiniabactin, ABC transporter, antimicrobial efflux, siderophore, mobile genetic element, integrative conjugative element

## Abstract

The accessory genomes of many pathogenic bacteria include ABC transporters that scavenge metal by siderophore uptake and ABC transporters that contribute to antimicrobial resistance by multidrug efflux. There are mechanistic and recently recognized structural similarities between siderophore importer proteins and efflux pumps. Here we investigated the influence of siderophore importer YbtPQ on antimicrobial resistance of *Klebsiella pneumoniae*. YbtPQ is encoded in the yersiniabactin cluster in a prevalent mobile genetic element ICEKp, and is also common in pathogenicity islands of *Escherichia coli* and *Yersinia* species, where yersiniabactin enhances virulence. Deletion of ICEKp increased the susceptibility of *K. pneumoniae* to all antimicrobials tested. The mechanism was dependent on the yersiniabactin importer YbtPQ and may involve antimicrobial efflux, since it was affected by the inhibitor reserpine. The element ICEKp is naturally highly mobile, indeed the accessory genome of *K. pneumoniae* is recognized as a reservoir of genes for the emergence of hospital outbreak strains and for transfer to other Gram-negative pathogens. Introduction of ICEKp, or a plasmid encoding YbtPQ, to *E. coli* decreased its susceptibility to a broad range of antimicrobials. Thus a confirmed siderophore importer, on a rapidly evolving and highly mobile element capable of interspecies transfer, may have a secondary function exporting antimicrobials.

## Introduction

ABC transporters in the accessory genomes of bacterial pathogens significantly influence both virulence and antimicrobial resistance. Siderophore importers scavenge metals from the host and efflux pumps export antimicrobials, and the presence of such transporters on mobile genetic elements is associated with both disease severity and treatment failure.

The nature of this transport is specific and unidirectional, due to the specific interactions between substrate and binding cavity, and the asymmetry of ATP-powered conformational changes through inward facing, closed and outward-facing forms. Broad-specificity multidrug efflux pumps are an apparent exception, and these have binding cavities with multiple sites that can interact with diverse antimicrobials (Du et al., 2018). Other rare examples of bispecific or multispecific unidirectional transport have been reported in bacteria and eukaryotes, such as siderophore export by multidrug efflux pumps, antibiotic entry through an asparagine importer, and chloroquine transport by a plasmodium peptide transporter (Hannauer et al., 2012; Smith et al., 2019; Shafik et al., 2020). By contrast, bidirectional ABC transporters that import one substrate and export another, are compatible with the mechanistic models but are unknown.

Siderophore importers are plausible candidates for such bidirectional transport since they have a spacious substrate binding cavity that might accommodate other molecules, and the structural organization is exporter-like (Arnold et al., 2020; Wang et al., 2020). Furthermore, in the context of hospital outbreak strains, a secondary function in antimicrobial export could provide a selective advantage.

The yersiniabactin siderophore cluster is prevalent and spreading in *Klebsiella pneumoniae* (Lam et al., 2018a,b) and was found in integrative and conjugative elements known as ICEKp1 or ICEKp3 in approximately half the *K. pneumoniae* clinical isolates tested in a recent United Kingdom and global study (Farzand et al., 2019). This cluster is also common in pathogenicity islands in *Escherichia coli* and *Yersinia* where it enhances virulence (Schubert et al., 2004; Lawlor et al., 2007; Perry and Fetherston, 2011; Koh et al., 2017).

**FIGURE 1:**
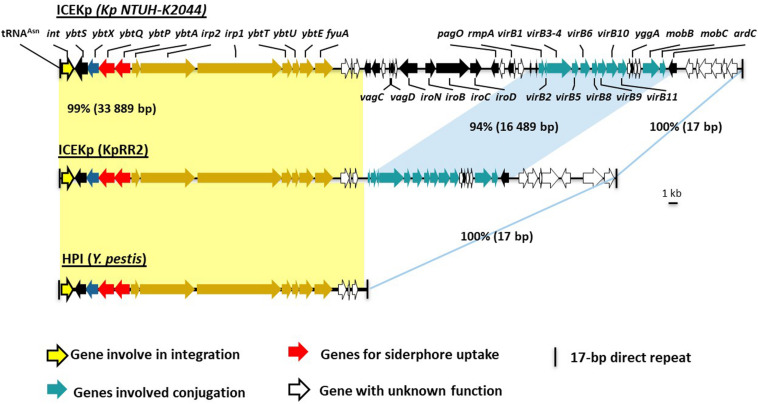
Comparison of ICEKp in the study strain KpRR2 with the first characterized ICEKp in strain NTUH-K2044, and with the yersiniabactin gene cluster in *Yersinia pestis*. The first characterization of ICEKp in *K. pneumoniae* was in strain Kp NTUH-K2044 (Lin et al., 2008). Yellow shading indicates the synteny and 99% DNA identity between the yersiniabactin gene cluster in the study strain KpRR2 and that in *Yersinia pestis* (AF091251) High Pathogenicity Island (HPI). The genes cluster responsible for conjugation are indicated by blue shading region, these share 94% identity.

**FIGURE 2:**
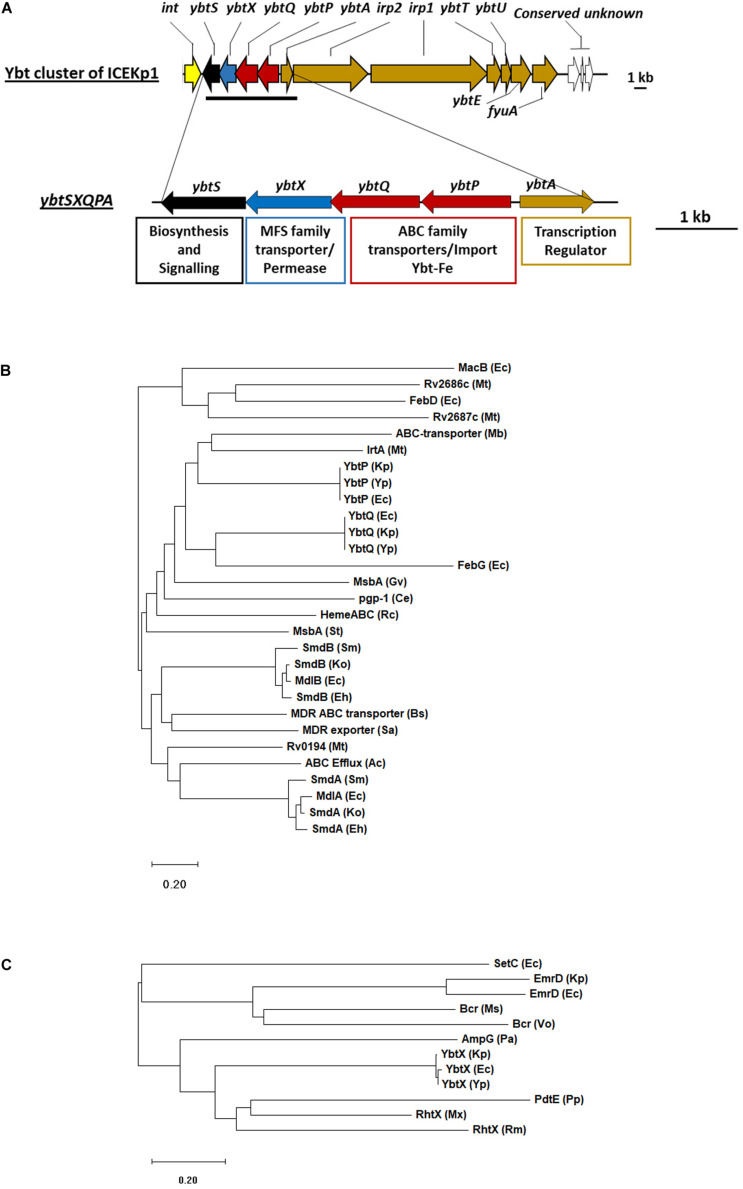
The cargo genes of ICEKp from *K. pneumoniae* KpRR2 are an iron siderophore biosynthesis cluster (yersiniabactin) that includes an ABC transporter and an MFS family permease. **(A)** The entire cargo genes of ICEKp are shown, with annotation. The only transmembrane transporters amongst the cargo genes are in the four-gene operon: *ybtP-ybtS*. This operon is unique to yersiniabactin clusters and conserved with >80% amino acid identity in all organisms. The previously reported deletion strain ΔICE lacks the entire ICEKp (and therefore lacks the entire yersiniabactin cluster). **(B)** YbtP and YbtQ share 34% amino acid identity with each other, and form a heterodimeric transporter YbtPQ, homologous to ABC transporters of drugs, heme, lipid A and enterobactin. ABC transporters are recognized by their nucleotide binding domain: the C-terminal domain of YbtP and YbtQ (residues 342–556 and 344–558) share 29% and 31% identity with the nucleotide binding domain of MacB. **(C)** YbtX belongs to the major facilitator superfamily (MFS) and is a homolog of aerobactin and rhizobactin transporters, multidrug efflux transporters and peptidoglycan recycling transporters. Ce, *Caenorhabditis elegans;* Ec, *E. coli*; Mt, *Mycobacterium tuberculosis;* Gv, *Gloeobacter violaceus;* Ko, *Klebsiella oxytoca;* Kp, *K. pneumoniae*; Mb, *Methanobrevibacter*; Ms, *Mycobacterium smegmatis;* Mx, *Myxococcus xanthus;* Pa, *Pseudomonas aeruginosa;* Pp, *Pseudomonas putida;* Sa, *Staphylococcus aureus*; Rc, *Rhodobacter capsulatus;* Rm, *Rhizobium meliloti*; St, *Salmonella typhimurium;* Vo, *Vibrio orientalis;* Yp, *Yersinia pestis*. Functional annotations of proteins: AmpG (muropeptide MFS transporter), Bcr (MDR efflux), EmrD (MDR efflux), FebDG (ferric enterobactin transporter), IrtA (iron transporter), MacAB (MDR efflux), MdlAB (MDR efflux), MsbA (lipid ABC transporter), PdtE (inner membrane permease) Pgp-1 (multidrug resistance protein), SetC (sugar efflux system), SmdAb (MDR efflux), RhtX (rhizobactin transporter), Rv0194 (MDR ABC transporter), Rv2886c/Rv2887c (MDR efflux). Phylogenetic tree of amino acids sequence of ybt genes was inferred by maximum-likelihood method using MEGA10 (Kumar et al., 2018). Bootstrap values were calculated with 1000 replications. The bar shows changes observed between the two sequences; 0.20 means 20% amino acid changes.

The cargo genes of ICEKp1 vary, and the example studied here, referred to as ICEKp from a clinical isolate of *K. pneumoniae*, the yersiniabactin cluster is the only cargo (Lam et al., 2018a,b; Farzand et al., 2019). Annotation of ICEKp and the yersiniabactin cluster is provided (Figures 1, 2). Yersiniabactin bound to iron is imported by a heterodimeric ABC transporter YbtPQ encoded within the yersiniabactin cluster, as shown by biochemical and phenotypic assays (Bearden et al., 1997; Fetherston et al., 1999; Lawlor et al., 2005; Koh et al., 2017; Wang et al., 2020). Another putative transporter encoded in the cluster is, YbtX, a permease of the MFS (major facilitator) superfamily, whose function is unknown, as its knockout in *Y. pestis* affected neither secretion nor utilization of yersiniabactin (Perry and Fetherston, 2011).

Antimicrobial resistance of *K. pneumoniae* arises from the combination of RND (resistance-nodulation-cell division family) efflux pumps and antibiotic resistance genes in the core genome [1] plus resistance determinants encoded on mobile genetic elements [17, 18]. Systematic deletion of mobile genetic elements from a clinical isolate has proven their contribution to antimicrobial resistance and generated a strain that is tractable for study by safe genetic modification [17, 18] and has been used to study gene mobilization by conjugation [9]. An ICEKp deletion mutant of this strain [9], apparently showed increased susceptibility to antimicrobials (reported below) despite the lack of obvious efflux pumps or antibiotic resistance genes in ICEKp, leading us to select this system to investigate, by genetic manipulation, whether YbtPQ influences antimicrobial susceptibility of *K. pneumoniae* and *E. coli*. We found that YbtPQ was necessary and sufficient to confer a modest but significant reduction in susceptibility (increase in minimum inhibitory concentration) for a broad range of antimicrobials (all antimicrobials tested) on both organisms, even in the absence of the rest of the yersiniabactin cluster or external yersiniabactin. The effect was likely due to antimicrobial efflux, since it was blocked by the efflux pump inhibitor reserpine.

## Results

### *ICEKp* and Its Transporter Gene Cluster Influence the Antimicrobial Susceptibility of *K. pneumoniae*

The influence of ICEKp and its cargo genes on antimicrobial susceptibility was investigated by measuring growth inhibition of *K. pneumoniae* by antimicrobials on agar using Estrips (minimum inhibitory concentration assay) and disks (zone of inhibition assay). We used the available ICEKp deletion mutant ΔICE that lacks the entire ICEKp element (Farzand et al., 2019) and had been constructed in strain KpRR2, which was derived from clinical isolate HS11286 (Liu et al., 2012; Bi et al., 2015). We initially tested a frontline treatment, the β-lactam cephalosporin ceftazidime, plus antimicrobials from four additional classes to target cell wall biosynthesis, protein synthesis and transcription. The minimum inhibitory concentrations of ΔICE were significantly lower than that of KpRR2 for all five antimicrobials, i.e., ceftazidime, erythromycin, streptomycin, rifampicin, tetracyclin (*p* < 0.05, Table 1). A plasmid pSXPQA was constructed to reintroduce the yersiniabactin transporter gene cluster (five genes: *ybtS, ybtX, ybtP, ybtQ, ybtA*), and this plasmid restored the MIC to parental levels for all antimicrobials (Table 1).

**TABLE 1:** Minimum inhibitory concentrations (MIC) for *K. pneumoniae* KpRR2, the mutant ΔICE and complemented strain ΔICE + pSXPQA for five antimicrobials.

Strain	MIC for antibiotic μg/ml*: Mean (Range)				
	
	Tetracycline	Streptomycin	Erythromycin	Ceftazidime	Rifampicin
KpRR2	3.2 (2–4)	2.1 (1–4)	35.6 (22–46)	1.9 (1.5–2)	11.2 (8–12)
ΔICE	1.8 (1.5–2)	0.37 (0.2–0.5)	8.7 (6–12)	0.6 (0.5–0.75)	2.3 (1.5–3)
ΔICE + pSXPQA	3 (2.5–4)	1.9 (1–4)	32.2 (21–38)	2 (1.5–2.5)	11.8 (11–12)
Fold change	**1.8**	**5.7**	**4.1**	**3.2**	**4.9**
KpRR2 vs. ΔICE					

*range tested using E-test strips (5 replicates): tetracycline, erythromycin, ceftazidime 0.016–256 μg/ml, streptomycin 0.064–1024 μg/ml, rifampicin 0.002–32 μg/ml.

**FIGURE 3:**
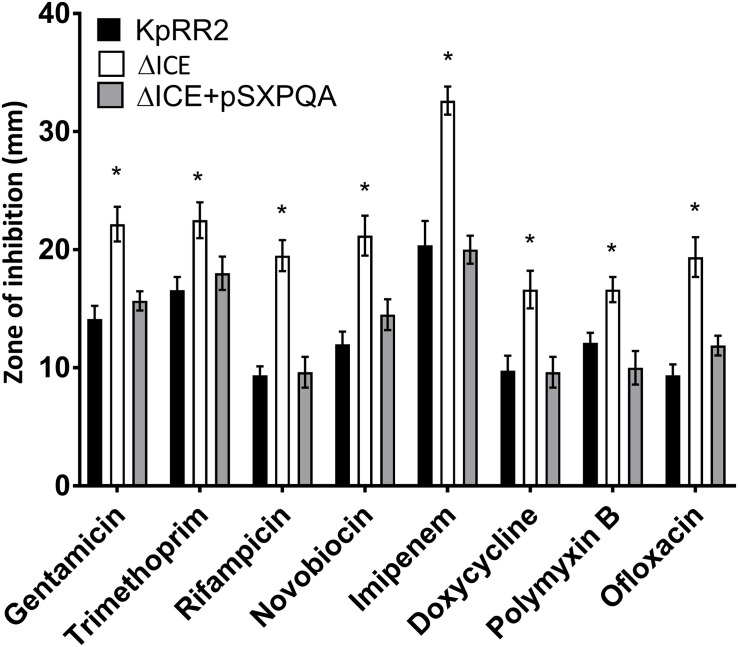
Deletion of the integrative and conjugative element ICEKp from *K. pneumoniae* rendered it more sensitive to multiple classes of antimicrobial, and the effect could be reversed by reintroduction of the yersiniabactin transporter gene cluster. A deletion mutant lacking the entire ICEKp element, ΔICE, had a significantly larger zone of inhibition than the parent strain KpRR2 for all antimicrobials tested. Reintroduction of the yersiniabactin transporter gene cluster on plasmid pSXPQA restored the zone size to that of the parental strain. Data show the mean and standard deviation of three replicates. The quantity of antimicrobial per disk was: gentamicin 10 μg, trimethoprim 5 μg, rifampicin 5 μg, novobiocin 30 μg, imipenem 10 μg, doxycycline 30 μg, polymyxin B 300 μg, ofloxacin 5 μg. Each strain was compared with the parent strain by Student’s *t* test, and significant differences in zone diameter were indicated with an asterisk. *p < 0.05.

Some proteins that transport antimicrobials are specific for a particular class of antimicrobial, while others are polyspecific (Du et al., 2018). To study larger numbers of antimicrobials, we used agar disk-diffusion assay, which is the official susceptibility test used in many clinical laboratories due to its simplicity, low cost, and suitability for high throughput (Balouiri et al., 2016). We verified the change in susceptibility to rifampicin and tested an additional frontline treatment (the β-lactam carbapenem imipenem), an additional example each of a tetracycline (doxycycline), and an aminoglycoside (gentamicin), two DNA gyrase inhibitors (ofloxacin and novobiocin) and a treatment of last resort (cationic peptide polymyxin B or colistin). For all eight antimicrobials, ΔICE was more sensitive than KpRR2 (significantly larger zone of inhibition, *p* < 0.05, Figure 3) whereas the plasmid-complemented mutant was not significantly different from the parent strain.

### *ICEKp* and the Transporter Gene Cluster Reduce Antimicrobial Susceptibility by Enhancing Antimicrobial Efflux

**FIGURE 4:**
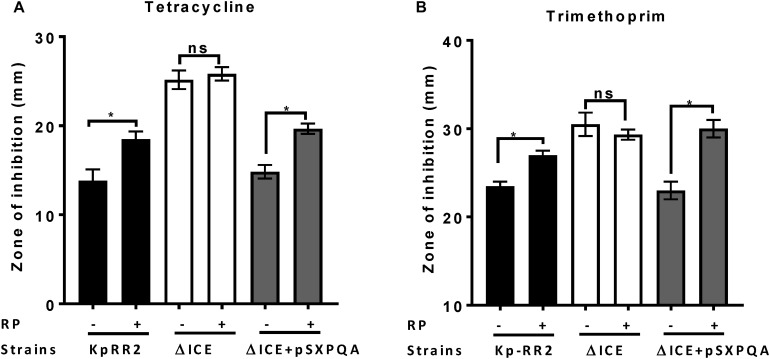
Efflux pump inhibitor reserpine increased the susceptibility of *K. pneumoniae*, but not the ICEKp knockout, to antimicrobials. Addition of reserpine (RP, 50 μg/ml) significantly increased the diameter of the zone of inhibition of study strain KpRR2 and the plasmid-complemented mutant ΔICE + pSXPQA for trimethoprim **(A)** and tetracycline **(B)**. By contrast, the presence (+) or absence (-) of reserpine had no significant effect on the zones of inhibition of the knockout strain ΔICE. Trimethoprim was used at 5 μg per disk and tetracycline at 25 μg per disk. Data are the mean and standard deviation of three replicates. *indicates *p* < 0.05 using Student’s *t* test. “ns” indicates *p* > 0.05. The assay was also performed with other antimicrobials giving similar results (Supplementary Figure 1).

ABC transporters that efflux antimicrobials can be blocked by the inhibitor reserpine (Schmitz et al., 1998; Dhanarani et al., 2017). To determine whether ICEKp affects antimicrobial susceptibility by causing efflux of antimicrobials, we repeated the disk diffusion assay using reserpine (Figure 4). Reserpine significantly enhanced the susceptibility of the parental strain and the complemented strain (*p* < 0.05) but not the mutant strain to tetracycline and trimethoprim (Figure 4) and other antimicrobials (Supplementary Figure 1), which would be consistent with the effects of ICEKp and the transporter plasmid acting through an efflux mechanism.

### The ABC Transporter YbtPQ Alone Is Sufficient to Reduce Antimicrobial Susceptibility of *K. pneumoniae* ΔICE

**FIGURE 5:**
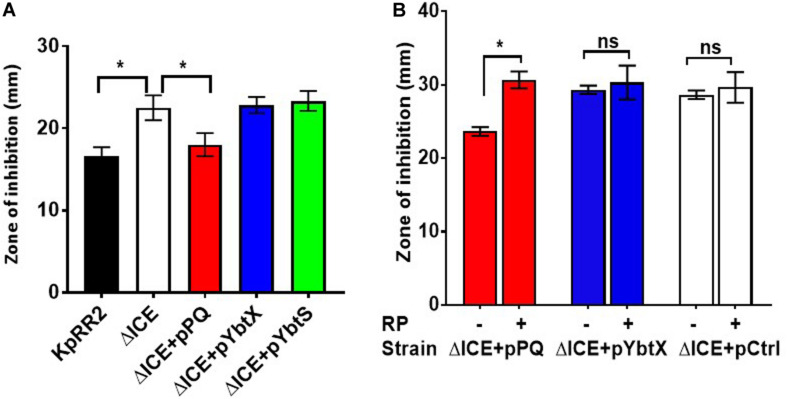
The yersiniabactin importer YbtPQ reduced antimicrobial susceptibility of *K. pneumoniae* ICEKp mutant and the effect of YbtPQ was reversed by reserpine. **A** The enhanced trimethoprim susceptibility (larger zone of inhibition) of mutant ΔICE was fully complemented by plasmid pPQ, encoding transporter YbtPQ. Plasmids pYbtX and pYbtS did not complement the defect of ΔICE (no significant change in zone of inhibition compared to ΔICE). Trimethoprim was used at 5 μg per disk. **B** The enhanced imipenem susceptibility of mutant ΔICE was fully complemented by plasmid pPQ, encoding transporter YbtPQ, but not by the vector control, pCtrl. The effect of plasmid pPQ on imipenem susceptibility was reversed by the addition of reserpine (RP) 50 μg/disk). Data are the mean and standard deviation of at least three replicates. *indicates *p* < 0.05 using Student’s *t* test. The effect of plasmid YbtPQ on antimicrobial susceptibility was also tested with additional antimicrobials (Supplementary Figure 2) and the effect of YbtPQ on antimicrobial susceptibility in the presence/absence of reserpine was additionally tested by MIC assay (Table 2).

Separate plasmids were constructed to determine which component(s) of the yersiniabactin transporter cluster influence antimicrobial susceptibility. *ybtPQ* and *ybtX* were chosen since these encode transmembrane proteins, and *ybtS* was chosen since it may be cotranscribed with *ybtPQ* and *ybtX*. Reintroducing the YbtPQ transporter using plasmid pPQ was necessary and sufficient to significantly reduce the zone of inhibition of ΔICE, such that it matched the parent strain for trimethoprim (Figure 5) and other antimicrobials (Supplementary Figure 2), whereas plasmids encoding YbtX or YbtS caused no significant change. Importantly, when ΔICE carrying plasmid pPQ was tested in the presence and absence of reserpine, we observed that the reduction in antimicrobial susceptibility conferred by *ybtPQ* was reversed by reserpine (Figure 5B and Table 2). Since YbtPQ reversed the changes in antimicrobial susceptibility caused by ICEKp deletion, we did not investigate the expression level or putative functions of YbtX and the function(s) of this putative transporter remain unknown.

**TABLE 2:** Minimum inhibitory concentrations (MIC) for *K. pneumoniae* mutant ΔICE containing control plasmid or plasmids encoding YbtPQ or YbtX in the presence or absence of reserpine (RP).

Strain	MIC for antibiotic μg/ml*: Mean (Range)			
	
	Tetracycline No RP	Tetracycline + RP	Streptomycin No RP	Streptomycin + RP
ΔICE + pCtrl	2.1 (1.5–2.8)	1.6 (1.5–2)	0.51 (0.3–0.75)	0.43 (0.3–0.5)
ΔICE + pPQ	4.2 (3–5.5)	2.1 (1.5–3)	1.9 (1.8–2)	0.67 (0.62–0.7)
ΔICE + pYbtX	1.8 (1.7–2)	1.6 (1.5–1.8)	0.66 (0.5–0.75)	0.38 (0.3–0.5)

*range tested using E-test strips (3 replicates): tetracycline 0.016–256 μg/ml, streptomycin 0.064–1024 μg/ml.

### Transfer of the Yersiniabactin Importer YbtPQ to *E. coli* Reduced Antimicrobial Susceptibility by an Efflux Mechanism

**FIGURE 6:**
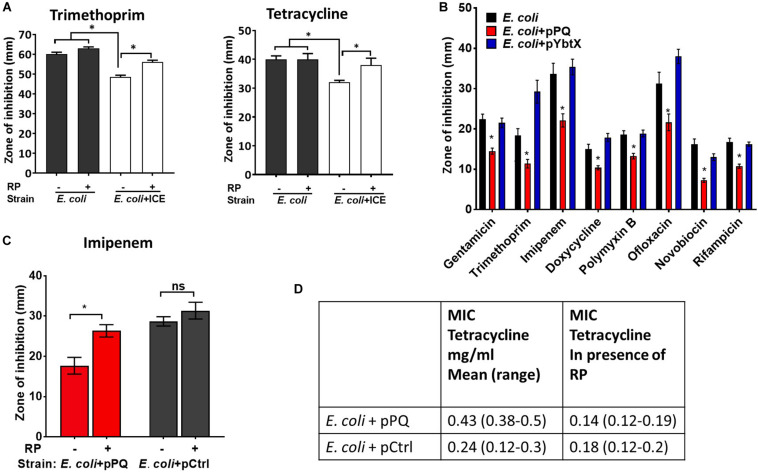
Antimicrobial susceptibility of *E. coli* was reduced by ICEKp or by YbtPQ alone. **(A)** Transconjugant *E. coli* + ICE carrying the ICEKp was significantly less sensitive than *E. coli* to trimethoprim and tetracycline in a disk diffusion assay (25 and 10 μg per disk, respectively). Inhibition of efflux using reserpine (50 μg/disk) significantly increased the susceptibility of the transconjugant to each antimicrobial. Reserpine (RP) had no significant effect on the susceptibility of parental *E. coli* to either antimicrobial. **(B)** *E. coli* carrying plasmid pPQ, encoding YbtPQ, were significantly less susceptible to all antimicrobials tested, whereas control plasmid pYbtX had no significant effect on antimicrobial susceptibility. From left to right, disks contained 10 μg, 5 μg, 10 μg, 30 μg, 300 units, 5 μg, 30 μg, or 5 μg of antimicrobial. **(C)** The effect of YbtPQ on susceptibility to imipenem was reduced by reserpine. pCtrl indicates a control plasmid without *ybtPQ* (vehicle only control). Data are the mean and standard deviation of six replicates. Strains and conditions were compared by Student’s *t* test. * indicates *p* < 0.05. **(D)** The effect of YbtPQ on the minimum inhibitory concentrations (MIC) of *E. coli* for tetracycline was determined using E-test assay in the presence or absence of reserpine (RP 50 μg/ml) in triplicate (range tested was 0.016–256 μg/ml).

ICEKp transfers efficiently from *K. pneumoniae* to *E. coli* by conjugation (Farzand et al., 2019). Transconjugant *E. coli* were produced and were significantly less sensitive than parental *E. coli* HB101 to tetracycline and trimethoprim (Figure 6A). Mirroring the results in *K. pneumoniae*, the efflux inhibitor reserpine abrogated the effect of ICEKp on antimicrobial susceptibility in *E. coli* (Figure 6A), and the plasmid encoding YbtPQ was sufficient to significantly reduce antimicrobial susceptibility (Figure 6B). The effect of YbtPQ was broad, since antimicrobials from diverse classes were chosen, and the of zone of inhibition was significantly reduced for all tested (Figure 6B). Reserpine significantly reduced the effect of YbtPQ on antimicrobial susceptibility (Figures 6C,D).

## Discussion

While there is considerable progress in conjugating siderophores with antimicrobials for ‘Trojan horse’ delivery of antimicrobials via siderophore uptake systems (Klahn and Bronstrup, 2017), the promiscuous ability of siderophore transporters to transport unmodified antimicrobials has yet to be determined. Here we demonstrate that the yersiniabactin cluster of *K. pneumoniae* affects antimicrobial susceptibility via the transporter YbtPQ, and this trait can be transmitted by conjugation to *E. coli* and therefore potentially other pathogenic *Enterobacteriaceae*. This suggests that two selective pressures, iron acquisition and antimicrobial resistance, might drive the acquisition, spread and evolution of elements carrying the yersiniabactin cluster. Notably, the same selective advantage may also apply to other siderophore gene clusters.

Direct efflux of antimicrobials by YbtPQ is the simplest explanation of the effects of YbtPQ on antimicrobial susceptibility of *K. pneumoniae* and *E. coli.* However, this is the first data indicating that any ABC transporter might be a bifunctional importer and exporter and will require further investigation into the potential activity and the mechanism. The structure of the inward-open conformation of the YbtPQ transporter is consistent with antimicrobial access to the cavity, which could lead to transport by the measured basal ATPase activity, or by antimicrobial-enhancement of ATP binding and hydrolysis (Wang et al., 2020). YbtPQ is thought to span the inner membrane, so any antimicrobial efflux from the periplasm would necessarily involve direct or indirect coupling to an outer membrane protein. Apart from the putative efflux activity of YbtPQ, alternative explanations for its effect on antimicrobial susceptibility could include YbtPQ-induced changes in gene expression or YbtPQ-catalyzed transport of other molecules. KpRR2 ΔICE and *E. coli* HB101 make only one siderophore, enterobactin, which is structurally and chemically dissimilar from yersiniabactin.

The reduction in antimicrobial susceptibility conferred by YbtPQ was broad: susceptibility was reduced for 13 out of 13 tested antimicrobials, and for some of antimicrobials like imipenem, the deletion of YbtPQ changed *K. pneumoniae* from resistant to sensitive according to the breakpoint in EUCAST (European Committee on Antimicrobial Susceptibility Testing 2020) (Kronvall et al., 2011). Broad specificity is a feature of some antimicrobial drug transporters, for example AcrB, an RND efflux pump (Yu et al., 2003), which reduces susceptibility to all antimicrobial classes tested in this study, and 10 out of 13 of the specific antimicrobials. As mentioned above, introduction of YbtPQ could affect antimicrobial susceptibility by multiple mechanisms including changes in expression of porins and transporters, therefore the broad reduction in antimicrobial susceptibility conferred by YbtPQ could reflect specificity of YbtPQ itself or other proteins and processes that YbtPQ affects. The inhibitor reserpine, which was used to test whether reduced antimicrobial susceptibility could be due to antimicrobial efflux, inhibits ABC, RND, and MFS transporters (Shaheen et al., 2019).

The yersiniabactin cluster is one of the key virulence associated factors reported in surveillance studies of outbreaks and spread of *K. pneumoniae* and here we demonstrate a potential selective advantage for this cluster in the presence of antimicrobials. Given that expression of the cluster is upregulated in infection models when iron availability is limited, this advantage could be significant in patients infected with *K. pneumoniae* who are receiving antibiotic treatment.

Other siderophore transporters from related or unrelated clusters might similarly influence antimicrobial resistance. The virulence enhancing piscibactin plasmids, carrying a YbtPQ homolog, are transmissible between species and enhance virulence of the economically fish aquaculture pathogen, *Photobacterium damselae* subsp *piscidia* (Osorio et al., 2015; Thode et al., 2018).

The facile movement of a virulence determinant from a multidrug resistant clinical isolate to another Gram-negative bacterium, with associated reduction in antimicrobial susceptibility, highlights the complex threat posed by the evolution and spread of drug resistance loci and drug resistant pathogens.

## Materials and Methods

### Bacterial Strains and Plasmids

*Klebsiella pneumoniae* and *E. coli* were cultivated in accordance with biosafety and institutional safety procedures using LB (Luria-Bertani or Lysogeny broth) or Luria agar. When needed for selection or maintenance of plasmids, chloramphenicol was added at 30 μg/ml. Reserpine was purchased from Fisher, dissolved in DMSO (dimethyl sulfoxide), filter sterilized, and added to agar at 50 μg/ml when indicated (final 0.1% DMSO).

*K. pneumoniae* KpRR2 was derived from clinical isolate HS11286 in a previous study (Bi et al., 2015). The GenBank Accession number for the HS11286 chromosome is CP003200. The chromosome sequence includes the *asn* tRNA-associated mobile genetic element ICEKp. ICEKp is also deposited separately as ICE*Kpn*HS11286-1 in the ICEberg database, ID 180 (Bi et al., 2012). Strain ΔICE was derived from KpRR2 previously by deletion of the entire ICEKp (Farzand et al., 2019).

### Filter Mating for Introduction of ICEKp to *E. coli* by Conjugation

A plasmid pOriT containing the chloramphenicol resistance cassette and origin of transfer (Farzand et al., 2019) was introduced to KpRR2 to act as a selectable marker for conjugation. Filter mating was used as described previously to transfer ICEKp with pOriT from KpRR2 to *E. coli* HB1010. Transconjugants were selected on LA with streptomycin 50 μg/ml and chloramphenicol 30 μg/ml (Farzand et al., 2019). PCR was used to confirm the presence of ICEKp and to verify the species using an *E. coli*-specific primer pair. Three transconjugants, termed *E. coli* + ICE, had equivalent phenotypes and were used in parallel for all experiments. Strain genotypes and primer sequences are listed in Supplementary Tables 1, 2.

### Construction of Complementation Plasmids

Plasmids pSXPQA, pPQ, pYbtX, and pYbtS were constructed by PCR amplification of the named gene(s) and cloning using the HD infusion cloning method (Raman and Martin, 2014) into plasmid pACYC184 (Chang and Cohen, 1978; Rose, 1988). Plasmid pSXPQA contained the five genes KPHS_34610-KPHS_34650 (*ybtS*-*ybtA* respectively) with their native promoter. The other three plasmids pPQ, pYbtX, and pYbtS, used the *crp* promoter. A control vector pCtrl was produced from pPQ by replacing *ybtPQ* with non-coding sequence of matching length taken from pOriT. In all cases, inserts replaced the tetracycline marker gene. Plasmid construction was verified by sequencing. Plasmids were introduced to *K. pneumoniae* and *E. coli* by electroporation (0.2 cm cuvette, 25 kV/cm, 25 μF, 200 Ω). The sequences of genes *ybtS* to *ybtA* are available in GenBank Accession number NC_016845.1 (Gene ID: 11848490, 11848491, 11848492, 11848493, and 11848494).

### Antibiotic Susceptibilities by Disk Diffusion and E-test Method

Colonies from overnight LA plate cultures were picked and suspended in sterile 0.9% NaCl. The turbidity of the suspension was adjusted to match McFarland 0.5 standard (∼0.8 at OD_600_). The suspension was spread evenly on Mueller Hinton agar within 15 min of preparation using a sterile cotton swab to create a semi-confluent growth. The inoculum was allowed to dry for 10 min before applying the antibiotic disks or E-test strips then the plates were incubated at 37^*o*^C for 24 h. Disks and E-test strips containing antimicrobial were purchased from Oxoid and BioMerieux respectively. Any plates with uneven growth were discarded. The zone of growth inhibition around the disks was measured in mm and results are reported as the mean and standard deviation of three or more independent experiments, for which each experiment had three technical replicates. Representative images are provided in Supplementary Figure 3. For E-test, the MIC (μg/ml) was read from the scale on the E-test strip where the symmetrical inhibition ellipse edge intercepts the strip and results are reported as the mean of five independent replicates.

## Data Availability Statement

The raw data supporting the conclusions of this article will be made available by the authors, without undue reservation.

## Author Contributions

RF designed the study, performed the experiments, analyzed the data, and wrote the manuscript. KR designed the study. MB, PF, GM, and MO analyzed the data. HO’H analyzed the data and wrote the manuscript. All authors contributed to the article, reviewed the manuscript and approved the submitted version.

## Conflict of Interest

The authors declare that the research was conducted in the absence of any commercial or financial relationships that could be construed as a potential conflict of interest.
