# Supplementary material for: A Virulence Associated Siderophore Importer Reduces Antimicrobial Susceptibility of Klebsiella pneumoniae
Source: Front Microbiol. 2021 Jan 28;12:607512. doi: 10.3389/fmicb.2021.607512 (PMC7876324; doi:10.3389/fmicb.2021.607512)
Supplement: Supplementary file 1 [file Data_Sheet_1.pdf]

Supplementary Material

Supplementary Table 1. Strains used in this study.

Strain	Feature	Reference
KpRR2	$\Delta bla_{KPC-2}\Delta$*KPHS_p300510-KPHS_p300880* ($\Delta bla_{KPC-2}\Delta$MDR)	[1]
ΔICE	$\Delta bla_{KPC-2}\Delta$*KPHS_p300510-KPHS_p300880* ($\Delta bla_{KPC-2}\Delta$MDR) ΔICE	[2]
E. coli HB101	Lacks the K-12 restriction-modification system (recA13 mutation) and has streptomycin resistance	[3]
E. coli+ICE	*E. coli* HB101+ICEKp (transconjugant)	This Study

Supplementary Table 2. Primers used in this study

Name	Primer sequence (5' to 3')	Use
VirB1_F	ATGCTTTCCACCACAGC	Specific to ICEKp; used to check transconjugants;
VirB1_R	TTATTCCTCCTCCTCACGG	
Int_F	TGTTCATTCGCTCCAGTGA	
Int_R	GGGTTATGGTCGCCGGGGA	
fyuA_F	CCTTCCCTTCCGGTTCGT	
fyuA_R	GCTCTTACCCTGGTCGCC	
irp1_F	CAGAAACGTGGCTCGACAAC	
irp1_R	CTTCGATGACTGCCTGTTGC	
EglmS-F	GATGACGGTTTGTCACATGG	Specific to *E. coli* genome; used to check transconjugants
EglmS-R	TTGTATGTCTTCGCCGATCAG	
P184-F	ACTGGCCTCAGGCATTTGA	To amplify plasmid pACYC184 for cloning reaction
P184-R	GTGCCTGACTGCGTTAGC	
SXPQA-F	ACGCAGTCAGGCACCTTACCTCTCTGTGTTATTCC	To amplify ybt transporter operon for cloning
SXPQA-R	ATGCCTGAGGCCAGTGTATCCGGGCCTCTGTCA	
PQ-F	ACGCAGTCAGGCACGACCTGGTTATCTCCCTGTG	To amplify *ybtP* and *ybtQ* for cloning
PQ-R	ATGCCTGAGGCCAGTGTCGTCAACGTCAGCGGTT	
ybtS-F	ACGCAGTCAGGCACTCTCGATGAACCGACTGCC	To amplify *ybtS* for cloning
ybtS-R	ATGCCTGAGGCCAGTTGGACAGTCTGGTTGTGAGG	
ybtX-F	ACGCAGTCAGGCACTGTTATTCCCGGATCAATCAAT	To amplify *ybtX* for cloning
ybtX-R	ATGCCTGAGGCCAGTGTCTGGAGAGTGGGTGCA	

Plasmids used in this study

pSXPQA - pACYC184 containing ybtS, ybtX, ybtP, ybtQ, ybtA replacing the tetracycline marker gene and containing the native promoter that lies between ybtA and ybtQ

pPQ - pACYC184 containing ybtP and ybtQ in place of the tetracycline marker gene, downstream of the crp promoter

pYbtX - pACYC184 containing ybtX in place of the tetracycline marker gene, downstream of the crp promoter

pYbtS - pACYC184 containing ybtS in place of the tetracycline marker gene, downstream of the crp promoter

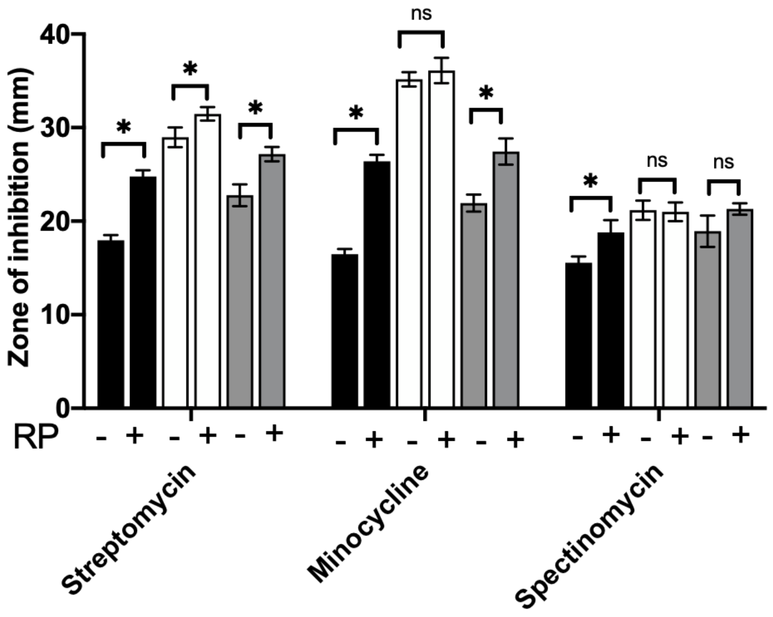

Figure S1. Efflux pump inhibitor reserpine (RP) increased the susceptibility of *K. pneumoniae*, but not the ICEKp knockout, to antimicrobials. Addition of reserpine (RP, 50 μg/ml) significantly increased the diameter of the zone of inhibition of study strain KpRR2 (black shading) for streptomycin, chloramphenicol and minocycline. By contrast, the presence (+) or absence (-) of reserpine did not significantly increase the zones of inhibition of the knockout strain ΔICE (white bars). Grey bars show the results for ΔICE complemented with plasmid pSXPQA. Antimicrobials were used at 10 μg, 30 μg and 30 μg per disc respectively. Data are the mean and standard deviation of three replicates. * indicates $p<0.05$ using student's t test. "ns" indicates $p>0.05$.

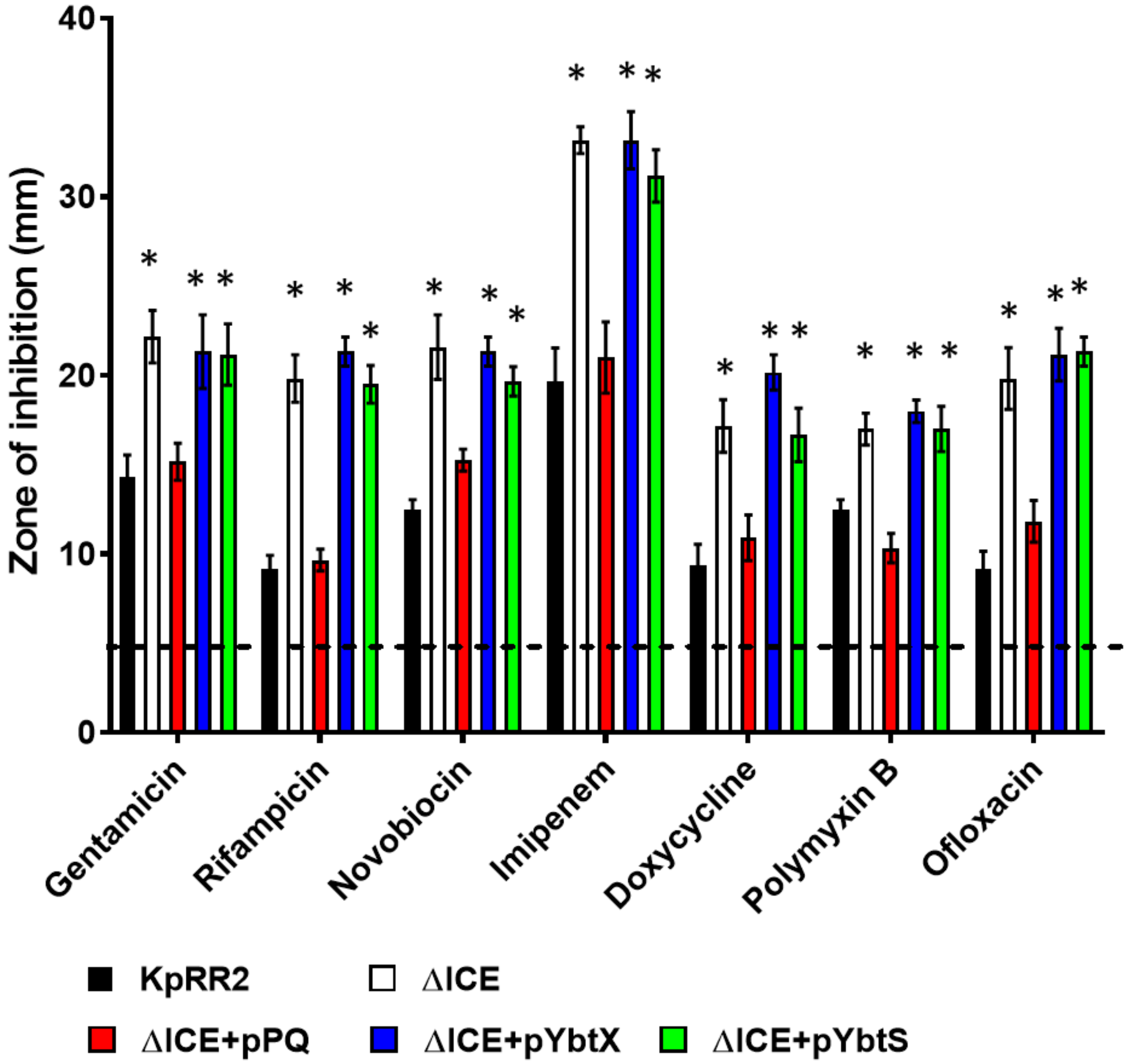

Figure S2. The yersiniabactin importer YbtPQ reduced antimicrobial susceptibility of *K. pneumoniae* ICEKp mutant. The enhanced trimethoprim susceptibility (larger zone of inhibition) of mutant ΔICE was fully complemented by plasmid pPQ, encoding transporter YbtPQ. Plasmids pYbtX and pYbtS did not complement the defect of ΔICE (no significant change in zone of inhibition compared to ΔICE). Antimicrobials were used at 10, 5, 30, 10, 30, 300 and 5 μg per disc. * indicates a significant difference in the zone of inhibition compared to the parental strain KpRR2 ($p<0.05$).

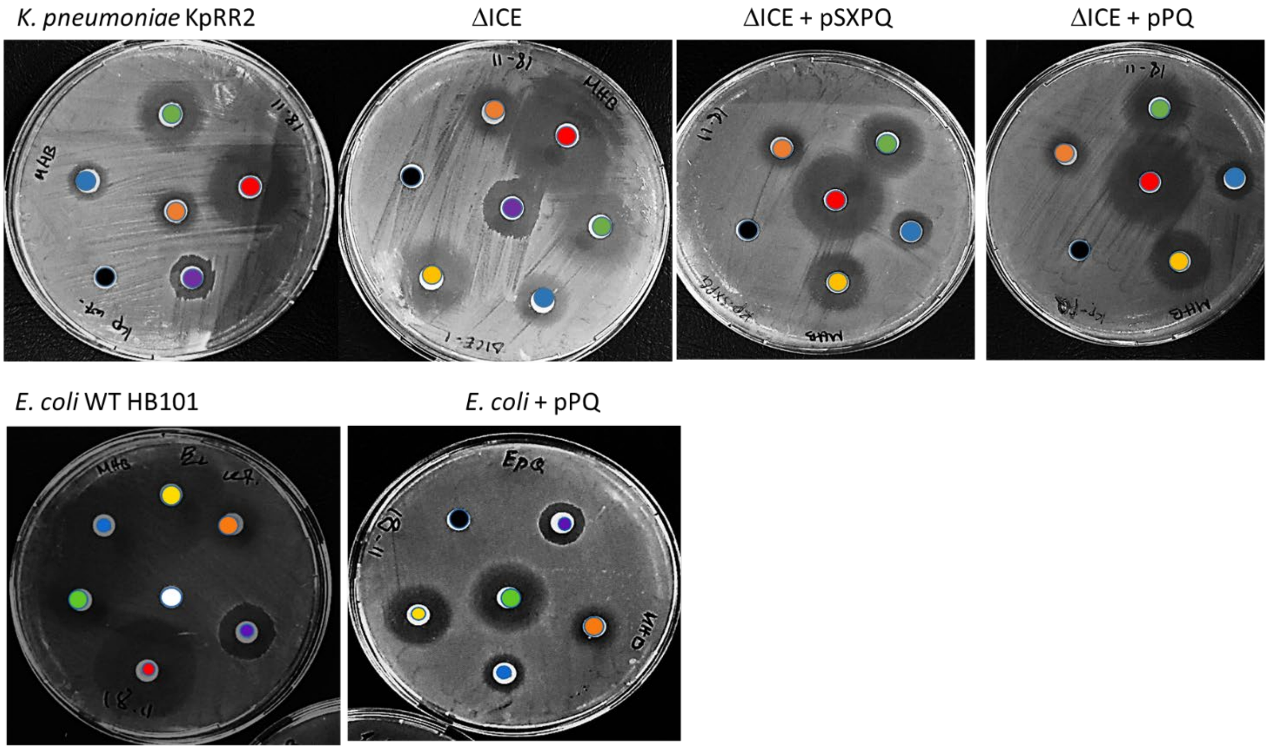

Figure S3. Representative images of disc diffusion assay of antimicrobial susceptibility. The diameter of zones of inhibition of bacterial growth was measured using at least 3 replicate plates for each antimicrobial for each strain. These images are representative examples of hundreds of plates used in this study. The antibiotic discs are colour coded: red contained imipenem, orange doxycycline, purple polymyxin B, black metronidazole, blue ofloxacin, green gentamicin, yellow trimethoprim, white ampicillin.

References

Bi, D., et al., *Mapping the resistance-associated mobilome of a carbapenem-resistant Klebsiella pneumoniae strain reveals insights into factors shaping these regions and facilitates generation of a 'resistance-disarmed' model organism.* J Antimicrob Chemother, 2015. **70**(10): p. 2770-4.

Farzand, R., et al., *ICEKp2: description of an integrative and conjugative element in Klebsiella pneumoniae, co-occurring and interacting with ICEKp1.* Scientific reports, 2019. **9**(1): p. 1-11.

Boyer, H.W., Roulland-dussoix, and Daisy, *A complementation analysis of the restriction and modification of DNA in Escherichia coli.* Journal of molecular biology, 1969. **41**(3): p. 459-472.
